# An Arched Micro-Injector (ARCMI) for Innocuous Subretinal Injection

**DOI:** 10.1371/journal.pone.0104145

**Published:** 2014-08-11

**Authors:** Yong Sung You, Chang Yeol Lee, Chengguo Li, Sung Ho Lee, Kibom Kim, Hyungil Jung

**Affiliations:** 1 Nune Eye Hospital, Seoul, Republic of Korea; 2 Department of Biotechnology, Yonsei University, Seoul, Republic of Korea; 3 Lumieye Genetics Co. Ltd., Seoul, Republic of Korea; University of Queensland, Australia

## Abstract

Several critical ocular diseases that can lead to blindness are due to retinal disorders. Subretinal drug delivery has been developed recently for the treatment of retinal disorders such as hemorrhage because of the specific ocular structure, namely, the blood retinal barrier (BRB). In the present study, we developed an Arched Micro-injector (ARCMI) for subretinal drug delivery with minimal retinal tissue damage. ARCMIs were fabricated using three major techniques: reverse drawing lithography, controlled air flow, and electroplating. In order to achieve minimal retinal tissue damage, ARCMIs were fabricated with specific features such as a 0.15 mm^−1^ curvature, 45° tip bevel, 5 mm length, inner diameter of 40 µm, and an outer diameter of 100 µm. These specific features were optimized via *in-vitro* experiments in artificial ocular hemispherical structures and subretinal injection of indocyanine green in porcine eye *ex-vivo*. We confirmed that the ARCMI was capable of delivering ocular drugs by subretinal injection without unusual subretinal tissue damage, including hemorrhage.

## Introduction

Irreversible visual loss can be caused by disruption of optic nerves in the retina resulting from retinal disorders such as retinal detachment, retinal vessel occlusion, and macular degeneration.[1]–[3] Due to the complex ocular structure, especially the blood retinal barrier (BRB), the subretinal space has become a target of interest for retinal treatment and thus has been used as an insertion site for drugs or stem cells in many medical procedures including transscleral injection,[4]–[5] subretinal implant,[6]–[7] and subretinal injection with vitrectomy.[8]–[9] All of these procedures require surgical intervention that can cause damage to the ocular tissue, and thus direct injection with a hypodermic needle having a small outer diameter is preferred over trans-scleral or subretinal injections.[4]–[9].

Flexible micro-cannula (39 gauge; 39 G) with an outer diameter of 120 µm have been introduced as subretinal injectors to reduce retinal tissue damage.[10] The linear form of this plastic cannula, however, is not sufficient to reduce the risk of unintended accidental punctures, which can cause retinal and choroidal hemorrhages during needle insertion in the subretinal region, which is located at the posterior region in the 70% spherical section of the eye and has a thickness of only several hundred micrometers. In addition, the flexibility of such micro-cannula devices is an inconvenience for injection into the soft subretinal space.[10] Although metallic shooter instruments have been developed for subretinal cell delivery, including a 20 gauge flattened 7 mm long metal tube bent at 25 degrees in consideration of the ocular structure, the size of this flattened tip was not able to penetrate the retina without causing retinal hemorrhage.[11] Recently, the tower microneedle (TM), a metallic hollow microneedle with a beveled tip, was developed for innocuous intravitreal drug delivery, and is one such application of hollow microneedles in the ocular research field.[12]–[13] Because TMs are designed to deliver drug into the intravitreal space through the sclera, they have enough strength to penetrate the outer ocular barrier of the sclera and even the conjunctiva. In addition, TMs have an outer diameter of 120 µm, and thus have the potential to be safe for subretinal injection. However, the straight shape of TMs remains an obstacle for its application in subretinal injections, because subretinal injection with minimal tissue damage is only possible using a curved needle/cannula shape that matches the spherical ocular structure. Although various subretinal injectors have been developed to minimize tissue damage, there is as of yet no single, ideal subretinal injector that satisfies the necessary criteria such as small tip diameter for minimal tissue damage, curved shape for delivering the drug into the ocular curve, and sufficient stiffness to minimize inconvenience during subretinal injection.

In this study, we present the arched micro-injector (ARCMI), which has a curvature of approximately 0.15 mm^−1^ in order to mimic the human ocular structure; this angle of curvature was found to be the most appropriate for ocular medical operations. In addition, the ARCMI has the smallest outer diameter (100 µm) of any existing ocular micro-injector, as well as sufficient stiffness to satisfy the conditions for an ideal subretinal injector. Subretinal injection in porcine eyes was performed *ex-*vivo using an ARCMI to deliver 10 µL of indocyanine green, which was chosen as a model drug. Indocyanine green was successfully injected into the subretinal space, where it remained without outflux to the vitreous cavity. Taken together, our results indicate that ARCMIs are associated with minimal damage to the retina and choroid tissue compared with existing methods.

## Materials and Methods

### Subretinal injection via an Arched Micro-injector (ARCMI)

**Figure 1:**
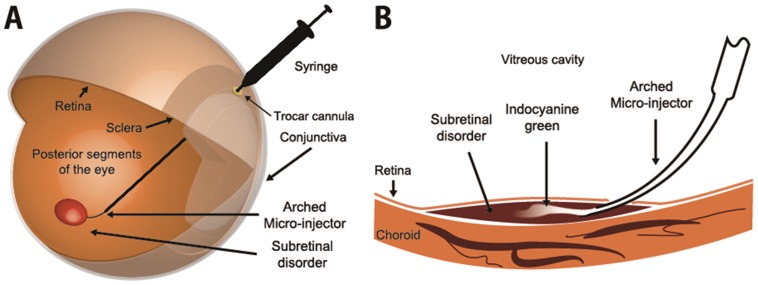
A comprehensive process of subretinal injection via the arched micro-injector (ARCMI). (A) The ARCMI penetrates the ocular curves by passing through a trocar cannula, which is used as a surgical aid for protection from ocular tissue damage in ophthalmology. The ARCMI can then be glided up to the target site at the retinal region of posterior segment of the eye. (B) Magnified image of subretinal insertion of indocyanine green via ARCMI. ARCMI slides along the retinal surface to reduce retinal tissue damage. Indocyanine green was injected into the subretinal target site.

The comprehensive process of subretinal injection via Arched Micro-injector (ARCMI) is illustrated in Figure 1A. The ARCMI can be inserted into the subretinal space in the posterior segments of the eye by passing through the side of the anterior ocular shell. During insertion of the ARCMI into the eye, a trocar cannula is used as a surgical aid to prevent damage to the ocular shells from the unanticipated force as well as to serve as a surgical passage. The ARCMI was designed to maintain its own curved shape even after penetration through the trocar cannula which was embedded in the anterior scleral near the pars plana. In the present study, ACRMIs were inserted into the subretinal space by gliding up to the target site for injection along the curved retinal surface in the posterior section of the eye as shown in the magnified cross sectional image (Figure 1B). Indocyanine green (Akorn, USA) was chosen as a model drug to easily detect the subretinal delivery and to inspect the retina hole created after subretinal insertion by the ARCMI without impairing the choroidal layer.

### Fabrication of the Arched Micro-injector (ARCMI)

**Figure 2:**
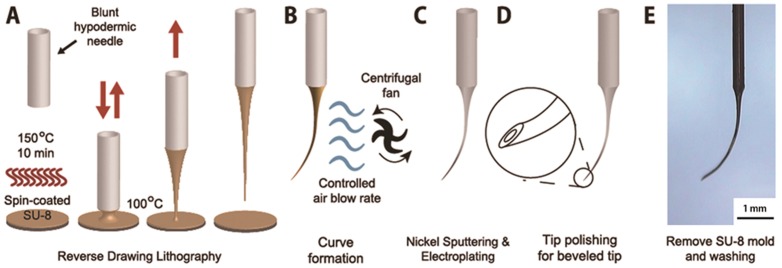
Illustration of the fabrication method for the arched micro-injector (ARCMI). (A) A straight SU-8 mold was formed at the end of blunt hollow metallic cannula (e.g., hypodermic needle) by reverse drawing lithography. (B) A flow of controlled air from a centrifugal fan produced the curved structure on the tip of the SU-8 mold of the ARCMI. (C) Hollow metallic walls on the mold surface were adopted by nickel sputtering and electroplating. (D) A hollow beveled tip was fabricated via tip polishing and removal of the SU-8 mold. (E) The hollow metallic structure of ARCMI formed after washing the mold.

As shown in Figure 2, each ARCMI was fabricated by modified reverse drawing lithography and a controlled air flow consisting of three major steps: solid micro mold fabrication by reverse drawing lithography, curvature adaptation on a solid micro mold, and formation of a hollow metallic structure formation. Reverse drawing lithography was performed by vertical drawing of heated SU-8 2050 viscoelastic material (Microchem, USA) using a 27 G hypodermic needle (Jung lim, Republic of Korea).[12] The drawing machine was created in house and was equipped with drawing (pulling) parts and heating parts. The drawing parts were located on the top of the drawing machine to allow for a vertical pulling motion for the fabrication of micromechanical mold from viscoelastic materials. The heating parts consisted of a round shaped metal plate to regulate temperature. Before drawing the solid micro mold, a 27 G hypodermic needle 1.5 inches in length with an outer diameter of 300 µm was adjusted on the drawing system. SU-8 2050 was coated on a 6-inch wafer to a thickness of 160 µm using a spin-coater (YS-100D, Republic of Korea) at a speed of 1000 rpm. The temperature was increased up to 150°C for 10 min, which was followed by solidification of SU-8 2050 at room temperature. Drawing was conducted vertically over a temperature range of 75 to 85°C at a rate of 1 mm/s to produce a solid mold diameter of 40±10 µm. The time required to raise the viscoelastic materials including SU-8 was such that it was directly associated with the length of fabricated solid molds. Specifically, linear solid micro molds with lengths of 5 and 10 mm were generated by pulling for 5 and 10 seconds, respectively.

To fabricate a variety of different curvatures of the solid micro mold, which was held vertically downwards at the drawing machine, a controlled flow of air was used. The airflow rate varied from 0, 3.2, 4.7 and 5.8 m/s for 10 minutes and was regulated with a perpendicular breeze produced from a centrifugal fan (Vivian, Republic of Korea). After curvature formation, a nickel seed layer for electroplating on the surface of ARCMI’s solid molds was produced by sputter coating (Leybol, Switzerland). Nickel electroplating was performed using an electroplating machine (Hwasung elc., Republic of Korea) with a 10 mA/mm^2^ current density for 2 hours resulting in a 30 µm nickel wall thickness, which was responsible for the outer diameter of the ARCMI. After electroplating, a tip bevel was modified with a polishing system constructed in our laboratory. The polishing system consisted of an 8 amp motor with a rubber bumper connected with a scotch stone. Electroplated ARCMI were placed onto a tilting system that held each ARCMI at a fixed angle. Polishing was performed by aligning each ARCMI over the scotch stone with motor rotating at a speed of 100 rpm. The resulting ARCMI had an inner surface curve to minimize tissue damage, and to easily identify successful drug delivery during subretinal injection. Hollow ARCMIs were produced by removing the solid micro mold of the ARCMI using an SU-8 remover (Microchem, USA) for 1 hour. The final ARCMI consisted of a hollow metallic micro-injector for subretinal injection, and after washing with distilled water, was connected to a hypodermic needle as shown in Figure 2E; this process automatically aligned the holes of the hypodermic needle and ARCMI concentrically. Because the diameter of solid mold corresponded to the inner diameter of ARCMI, we adjusted the solid mold diameter to 40±10 µm during reverse lithography process by controlling SU-8 thickness and the drawing temperature. The outer diameter of the ARCMI was determined by the inner diameter and various wall thickness values, which were controlled by altering the electroplating time and electric currents. In subsequent experiments, we used ARCMIs with specific dimensions as follows: length of 5 or 10 mm, inner diameter of 40 µm, outer diameter of 100 µm, and a bevel tip angle of approximately 45°.

### Curvature of the ARCMI

**Figure 3:**
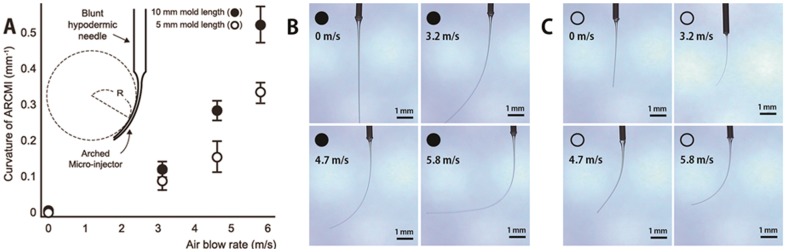
Different curvatures of SU-8 mold of Arched Micro-injector (ARCMI) produced by increasing airflow rate. (A) The ARCMI curvature was defined as the inverse of the radius of a circle fitting the inner ARCMI curve (Inset). The radius of this circle was measured by photo analysis with real-time microscope. ARCMIs were fabricated with different curvatures and lengths (A). Image of ARCMI molds with various curvatures for lengths of (B) 10 mm length and (C) and 5 mm. ARCMI curvatures depended on both the mold length and rate of airflow. Scale bar: 1 millimeter.

The curvature of the ARCMI, *k*, was defined as
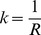
where *R* is the radius of curvature. An arbitrary circle was fitted with the curve of each ARCMI to measure the radius of curvature. A section of the circle (arc) was used as the reference point for taking the radius as shown in the inset of Figure 3A. Each curvature was calculated with computer based imaging software (Saram soft, Republic of Korea) using images captured with a brightfield microscope (Samwon, Republic of Korea).

### Measurement of the mechanical fracture force of ARCMIs

The mechanical fracture force of fabricated ARCMIs was determined using a force measurement machine (Zwick, Germany) at a rate of 1 mm/min. The fracture forces of ARCMI were measured at the first peak of the force measurement graph, with a unit of force measurement of 0.001 Newton (N). Vertical and transverse fracture forces were determined by axial alignment of the ARCMI vertically and transversely, respectively, in the testing apparatus. Various ARCMIs with curvatures of 0, 0.15 and 0.3 mm^−1^ and lengths of either 5 or 10 mm length were prepared for a combination of 6 separate samples and were used in the mechanical fracture force measurement test.

### *In vitro* Subretinal injection of ARCMI in an artificial eye

Subretinal insertions of ARCMI with various curvatures were conducted in an artificial ocular structure to determine the optimal curvature of ARCMI for innocuous subretinal injection. The artificial ocular structure was prepared by mimicking the human adult ocular region, which has a spherical shape with an inner diameter of 25 mm, using a custom hemispherical aluminum molding box prepared in house. The hemispherical structure of the artificial ocular curve was adopted as an ideal model to analyze needle insertion, because the target sites for subretinal injection are mainly located in the posterior segment of the eye. The artificial ocular structure was hemispherical and consisted of an artificial choroid layer and artificial retinal layer. The artificial choroid layer (hemispherical layer) with a thickness of 5 mm was obtained from the aluminum molding box after 20 mL of polydimethylsilane (PDMS, Sewang High Tech., Republic of Korea) was allowed to solidify at 80°C in an oven (JEIO TECH, Korea) for 24 hours. The artificial retinal layer consisted of an agarose gel (2 g/mL, Invitrogen, USA) poured along the inner surface of the artificial choroid layer and had an inner diameter of 23 mm and a thickness of 1 mm. In order to match the thickness of human retina, the agarose gel layer was dried for 24 hours at room temperature to reduce its thickness from 1 mm to 0.5 mm.

Ten microliters of indocyanine green (Sigma Aldrich, USA) were injected into the artificial subretinal region using ARCMIs with various curvatures of 0, 0.15, and 0.3 mm^−1^. The curved tip was used to glide along the inner surface of the artificial retina, which allowed the ARCMI to be carefully inserted into the artificial subretinal space with minimal intervention so as to reduce damage to the artificial retina. The insertion track was confirmed by cross sectional analysis with a brightfield microscope (Samwon, Republic of Korea).

### *Ex vivo* subretinal injection of ARCMI in sacrificed porcine eyes

All surgical procedures were approved by the Institutional Animal Care and Use Committees of Nune Eye hospital and Lumieye Genetics Co., Ltd (LG-IACUC-2011-1228) and were conducted by ophthalmologists at the Nune Eye Hospital using porcine eyes that were prepared within one day of sacrifice. Fresh porcine eyes used in this research were purchased from local market (Booil Co., Republic of Korea). The surgical procedure was performed using a standard 3-port pars plana vitrectomy, which is a common ophthalmology procedure.[14] After fixing the porcine eye (Seoulin Bio, Republic of Korea) on a stage with pins, three cannulas were inserted on the porcine ocular surface to model the connection between the porcine eye and surgical instruments such as light source needle, water supporter, and suction needle. A cannula was also inserted to prevent needle mobilization, which can cause unexpected damage to the outer ocular layers, including the sclera. The inside contents of the porcine eye (e.g., Hyaluronic acid) were extracted with a needle. Distilled water was passed into the eye through the inlet cannula and passing to the outlet cannula. The inside of the eye was brightened by a light source (Alcon Systems, USA). The circumference inside the eye was observed through the pupil by brightfield microscopy (Alcon Systems, USA).

For *ex vivo* experiments, each ARCMI was inserted into a cannula connected to the interior of the eye. The ARCMI was also connected to a 1 mL syringe (Korea vaccine Co. Ltd., Republic of Korea) attached to an air compressor (Alcon Systems, USA) through a transparent hose. The *ex *vivo test for subretinal injection consisted of needles with no curve (straight form), a large outer diameter (>200 µm), curved with bevel tip, curved without bevel tip, and were used to inject 10 µL of indocyanine green. A straight micro-injector lacking a bevel tip was used as a control for subretinal injection.

### Histological analysis of the retinochoriodal structure

Lastly, we performed histological analysis of porcine retina after subretinal injection using various ARCMI devices. Cryosections of porcine ocular tissue including the retina were obtained immediately after subretinal injection of indocyanine green with different ARCMI devices. Ten µm thick tissue slice of porcine retina tissue, including the subretinal injection hole resulting from ARCMI insertion, were obtained using a microtome (LEICA, Germany). Sections were also stained with Hematoxylin (Sigma Aldrich, USA) and Eosin (Sigma Aldrich, USA). Stained tissues were observed using a brightfield microscope (Olympus, Japan).

### Statistical Analysis

All data was represented as a mean value for at least three individual experiments or mean ±standard deviation. Significance determined by Student t-test (ρ<0.05).

## Results

### Fabrication of the arched micro-injector (ARCMI) for subretinal injection

Solid molds for ARCMIs with a variety of curvatures were fabricated using controlled airflow rates for different mold lengths (5 and 10 mm) as shown in Figure 3. As the airflow increased from 0 to 5.8 m/s, the curvature of ARCMI increased, and was more prominent for the 10 mm mold length compared to the 5 mm mold (Figure 3A). Specifically, the mold curvature for an ARCMI with a length of 10 mm was over 50% higher than an ARCMI 5 mm in length, reaching 69% at 3.2 m/s, 53% at 4.7 m/s, and 63% at 5.8 m/s. Images of the various curvatures obtained from the ARCMI mold are shown in Figure 3B. Curvature was not induced at a lower airflow rate of 0 m/s, whereas a hook shaped needle mold was produced for flow rates greater than 10 m/s (data not shown). In addition, after 10 minutes of air flow there was no further change in the shape of the ARCMI micro solid mold. The human ocular curvature is around 0.09 mm^−1^, which could be obtained with an airflow of 4 m/s.

### Mechanical properties of ARCMIs of various lengths

We next analyzed the mechanical fracture forces of ARCMIs with different curvatures of 0, 0.15, and 0.3 mm^−1^ and lengths of 5 and 10 mm. The inner- and outer diameters of each ARCMI were fixed as 40±10 µm and 100±10 µm, respectively. There was no distinguishable difference in axial fracture force between ARCMIs with different curvatures, because deformation of the needle occurred at equal axial forces. However, fracture forces decreased as the ARCMI length increased. Specifically, the axial and transverse fracture forces of ARCMIs 5 mm in length (n = 9) were found to be 2.1±0.4 N and 0.6±0.1 N, respectively, while the forces of ARCMIs 10 mm in length (n = 8) were 1.8±0.7 N and 0.5±0.1 N, respectively.

### Subretinal injection in an artificial eye using the ARCMI

**Figure 4:**
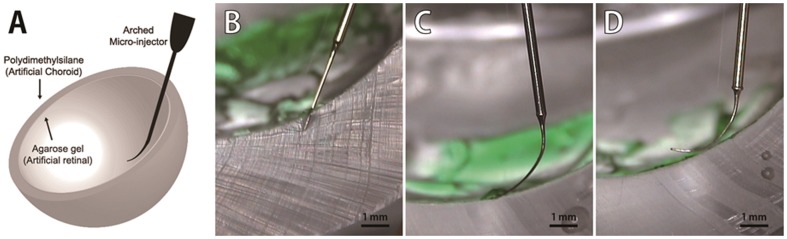
Subretinal injection of indocyanine green using the ARCMI in an artificial eye. (A) The artificial eye was hemispherical composed of two layers consisting of an artificial retina (agarose gel) layer and artificial choroid (polydimethylsilane; PDMS) layer. (B) A straight needle ARCMI (curvature = 0) was difficult to insert into the subretinal space without incurring tissue damage. (C) An ARCMI with a curvature of around 0.15 mm^−1^ could be inserted into the artificial subretinal space through the agarose gel layer without damaging the PDMS layer. (D) Insertion of a needle with a curvature over 0.3 mm^−1^ was difficult because of the large surface of contact area with the artificial retina (D). Scale bar: 1 millimeter.

Subretinal injection using the ARCMI was performed in an artificial hemispherical eye mimicking the human eye as shown in Figure 4A. To minimize retina and choroid tissue damage, ARCMIs 5 mm in length with curvatures of either 0, 0.15, or 0.3 mm^−1^ were inserted through the agarose gel (artificial retina) layer obliquely, but not the PDMS (artificial choroid) layer. The micro-injector with a 0 mm^−1^ curvature was difficult to insert into the artificial subretinal space without invasion into the PDMS layer during subretinal injection (Figure 4B). Conversely, the ARCMI with a 0.15 mm^−1^ curvature was able to insert into the artificial subretinal space without PDMS layer destruction (Figure 4C). The ARCMI with a 0.3 mm^−1^ curvature produced a large surface area of contact resulting in severe damage to the gel (artificial retina) (Figure 4D), while curvatures greater than 0.3 mm^−1^ were not only difficult to insert into the subretinal space, but also induced a wide surface area of contact with the retina.

### Subretinal injection into porcine eyes using ARCMI

**Figure 5:**
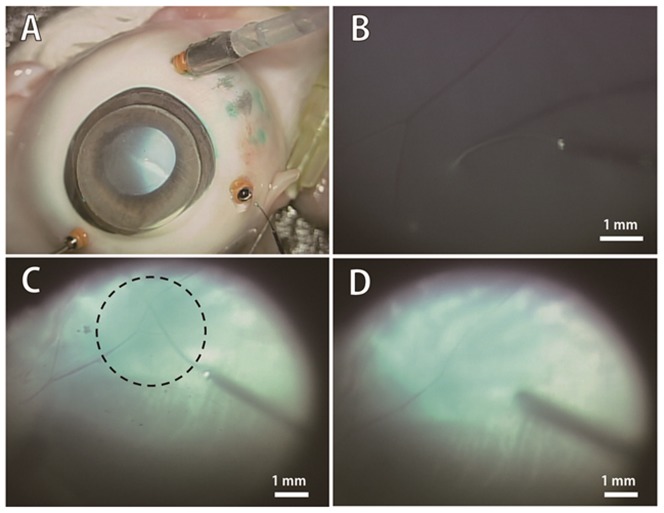
*Ex-vivo* test of subretinal injection of indocyanine green via ARCMI in porcine eye. (A) The *ex-vivo* test of the subretinal injection was performed using a standard 3-port pars plana vitrectomy, which is a widely used ocular surgical procedure for retinal disorder treatment. (B) All procedures were performed by ophthalmologists. The ARCMI reached on to the surface of the porcine retina, and the distance between retinal surface and ARCMI was determined using the shadow of the injector. (C) Indocyanine green with 10 µL was injected into subretinal space via ARCMI. (D) Subretinal injection of indocyanine green spread specifically in the subretinal space.

We next performed subretinal injections in porcine eyes to study whether ARCMIs can inject drugs into the subretinal space with minimal tissue damage in real eyes (Figure 5A). An ARCMI with a 0.15 mm^−1^ curvature, 5 mm length, and 45° bevel tip angle was applied onto the porcine retina surface obliquely (Figure 5B). The distance between the retinal surface and ARCMI could be determined using the shadow of the ARCMI tip. Indocyanine green (10 µL) could be smoothly injected via the ARCMI into the subretinal space with a pressure of 15 mmHg. Importantly, indocyanine green spread specifically in the subretinal space (dashed circle in Figure 5C), indicating successful subretinal injection with the ARCMI. Indocyanine green was predominantly present in the subretinal space after 5 minutes without fade away in vitreous cavity (Figure 5D).

### Histological analysis of retinal damage

**Figure 6:**
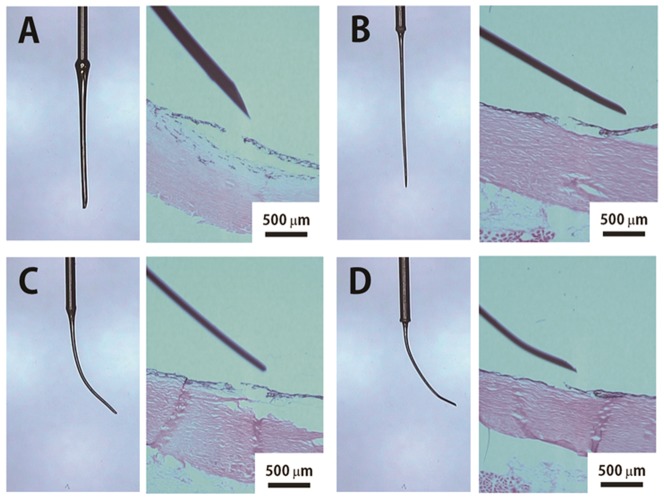
Histological analysis of porcine eyes after subretinal injection. Cryosection and tissue staining (Hematoxyline and Eosin) of the retina-choroid tissue of porcine was performed after subretinal injection with various tip form of Arched Micro-injector such as (A) no curved tip with large outer diameter (200 µm), (B) small outer diameter (100 µm) tip, (C) a 0.15 mm^−1^ curvature with 100 µm outer diameter and no beveled tip, and (D) a 45° bevel angle tip with 0.15 mm^−1^ curvature and 100 µm outer diameter. The straight tip caused damage to the retina as well as choroid surface. Lack of a beveled tip tore the retina surface resulting in a larger hole on the retina.

Although subretinal injection performed obliquely with the ARCMI reached the inner surface of the retina, a straight micro-injector with an outer diameter of 200 µm invaded the choroid layer as well as the retina to reach the subretinal space, producing a large hole on the retinal surface (Figure 6A). A micro-injector with a smaller outer diameter of 100 µm also was not well suited for making an innocuous subretinal injection without severe retinal tissue damage (Figure 6B). We also tested the effect of the bevel angle on retinal and choroidal damage using an ARCMI with a curvature of 0.15 mm^−1^. As shown in Figs. 6C and 6D, even if curvatures were adopted on the micro-injectors with the smaller outer diameter (100 µm), the degree of subretinal tissue impairment varied with the different needles resulting in a choroidal layer rip if a beveled tip was not present (Figure 6C).

## Discussion

Subretinal injection with minimal tissue damage is possible with a curved needle/cannula shape that matches the spherical ocular structure. Thus, for ideal subretinal drug injection into the curved subretinal region to minimize tissue damage, the key parameter is to fabricate a curve shape injector with a small diameter.[1] In this study, we propose the ARCMI, which was generated by controlled airflow over a thermosetting polymer (e.g. SU-8 photoresist) during the process of reverse drawing lithography (Figure 2). The degree of curvature depended on the rate of airflow and length of the mold (Figure 3A). Because length of the ARCMI mold relied on drawing speed and time, the combination of reverse drawing lithography technique and controlled airflow facilitated generation of ARCMIs with the desired appearance. Shorter length (5 mm) ARCMIs exhibited superior mechanical properties and were preferred over longer length (10 mm) as candidate for subretinal injectors, because mechanical fracture force of ARCMI as a function of stiffness is important to minimize inconvenience due to flexibility during subretinal injection. However, the fracture forces of ARCMI 10 mm in length indicated they still had sufficient stiffness to penetrate the layers of the retina layer, and even the choroid. Thus, the methods used to fabricate the various ARCMIs resulted in enough stiffness to penetrate into the subretinal region regardless of curvature or length (5 and 10 mm).

The effect of curvature on tissue damage was first analyzed using *in-vitro* experiments with an artificial ocular hemispherical structure, which was followed subretinal injection of indocyanine green in *ex vivo* porcine eye tests. We found that the optimum curvature of the ARCMI was 0.15 mm^−1^. As expected, straight micro-injectors caused severe damage to the choroid tissue, because the target site, the subretinal region, has a thickness of only 0.5 mm on the curved ocular structure. In addition, an ARCMI with a curvature of approximately 0.3 mm^−1^ was difficult to insert into the subretinal region and also induced a large contact area on the retina surface, which result in retina damage. The effect of curvature was confirmed by histological analysis after subretinal injection of ARCMI in the porcine eye. A straight micro-injector with a smaller outer diameter of 100 µm was also not appropriate for making innocuous subretinal injections without severe retinal tissue damage. Together, these results implied that the curvature of the injector is critical for avoiding choroidal invasion from the needle tip. In addition, we tested the effect of the presence and absence of a bevel during subretinal injection on tissue damage. Despite using a curved shape, ARCMIs without a bevel tore the retina surface resulting in a larger hole on the retina. Thus, only a curved and beveled tip minimized the hole on the retina without choroid tissue damage, because beveled tips decrease insertion force.[12].

Indocyanine green (10 µL) could be smoothly injected by an ARCMI with a 0.15 mm^−1^ curvature, 5 mm length, and 45° bevel tip angle onto the porcine retina surface obliquely with a pressure of 15 mmHg. Importantly, dye injected in this way spread specifically in the subretinal space. When the dye was injected into the vitreous cavity, no spreading was observed and the dye diffused quickly and diluted in the vitreous cavity. In addition, no out flux of dye from the injection site to the vitreous cavity was observed for 5 minutes after injection, indicating that indocyanine green was predominantly present in the subretinal space. The ARCMI device described in this study should facilitate local delivery of drugs to specific clinical point of the subretinal region, and thus simultaneously overcome limitation of current subretinal injectors such as small tip diameter for minimal tissue damage, curved shape for delivering the drug into ocular curve, and sufficient stiffness.

## Conclusions

To achieve innocuous subretinal injection, we developed the ARCMI, which was optimized for specific characteristics including a length of 5 mm, curvature of 0.15 mm^−1^, an outer diameter of approximately 100 microns, and a 45° beveled tip for minimal tissue damage during subretinal injection. Especially, the 0.15 mm^−1^ curvature of the ARCMI was a major factor allowing it to achieve minimal retinal tissue damage by fitting with the curvature of the human ocular surface. Our data suggest that the ARCMI is a suitable alternative to traditional subretinal injectors, such as hypodermic needles, for innocuous subretinal injection. Furthermore, ARCMIs may be useful for a number of different applications, including safe drug delivery with minimal tissue damage as well as cell delivery into specific organs such as the brain.
